# The Special Measures for Quality and Challenged Provider Regimes in the English NHS: A Rapid Evaluation of a National Improvement Initiative for Failing Healthcare Organisations

**DOI:** 10.34172/ijhpm.2022.6619

**Published:** 2022-04-27

**Authors:** Cecilia Vindrola-Padros, Jean Ledger, Melissa Hill, Sonila Tomini, Jonathan Spencer, Naomi J. Fulop

**Affiliations:** ^1^Department of Applied Health Research, University College London, London, UK.; ^2^Department of Targeted Intervention, University College London, London, UK.; ^3^NHS North Thames Genomic Laboratory Hub, Great Ormond Street Hospital, London, UK.; ^4^Nuffield Trust, London, UK.

**Keywords:** Low-Performing, High-Performing, Improvement, Interventions, Special Measures for Quality, UK

## Abstract

**Background:** There is limited knowledge about interventions used for the improvement of low-performing healthcare organisations and their unintended consequences. Our evaluation sought to understand how healthcare organisations in the National Health Service (NHS) in England responded to a national improvement initiative (the Special Measures for Quality [SMQ] and challenged provider [CP] regimes) and its perceived impact on achieving quality improvements (QIs).

**Methods:** Our evaluation included national-level interviews with key stakeholders involved in the delivery of SMQ (n=6); documentary analysis (n=20); and a qualitative study based on interviews (n=60), observations (n=8) and documentary analysis (n=291) in eight NHS case study sites. The analysis was informed by literature on failure, turnaround and QI in organisations in the public sector.

**Results:** At the policy level, SMQ/CP regimes were intended to be "support" programmes, but perceptions of the interventions at hospital level were mixed. The SMQ/CP regimes tended to consider failure at an organisational level and turnaround was visualised as a linear process. There was a negative emotional impact reported by staff, especially in the short-term. Key drivers of change included: engaged senior leadership teams, strong clinical input and supportive external partnerships within local health systems. Trusts focused efforts to improve across multiple domains with particular investment in improving overall staff engagement, developing an open, listening organisational culture and better governance to ensure clinical safety and reporting.

**Conclusion:** Organisational improvement in healthcare requires substantial time to embed and requires investment in staff to drive change and cultivate QI capabilities at different tiers. The time this takes may be underestimated by external ‘turn-around’ interventions and performance regimes designed to improve quality in the short-term and which come at an emotional cost for staff. Shifting an improvement focus to the health system or regional level may promote sustainable improvement across multiple organisations over the long-term.

## Background

**Key Messages**

** Implications for policy makers**

- Organisational improvement in healthcare requires substantial time to embed and requires investment in staff to drive change and cultivate quality improvement (QI) capabilities at different tiers.
- It is important to take into consideration the potential negative consequences of programmes such as Special Measures for Quality/challenged provider (SMQ/CP) such as the emotional impact on staff and the hospital’s ability to retain and recruit staff.
- Shifting an improvement focus to the health system or regional level may promote sustainable improvement across multiple organisations over the long-term.

** Implications for the public**

The study identified factors that can act as drivers for the improvement of hospital performance, leading to better care delivery for patients. We found that some of the organisational processes established as a result of the integration of quality improvement (QI) led to better governance arrangements and the development of strategies to improve patient safety. Staff engagement and an organisational culture that supports learning were identified as key components to sustainable improvement and the delivery of high quality care for patients. The study also pointed to the importance of considering improvement beyond individual organisations, to include the wide range of services and actors that patients might interact with at a regional level.

There is an internationally recognised need for transparent, integrated, and timely processes for identifying and addressing quality and patient safety issues across healthcare systems.^1^ Attention has been placed on failing healthcare organisations, their characteristics and the factors (internal and external) that might lead to low performance. These have included low leadership capability, lack of open culture, antagonistic external relationships (for instance, with other provider organisations),^2-4^ inadequate infrastructure, lack of a cohesive mission, and system shocks.^5^ A hierarchical culture and leadership focused on avoiding penalties and achieving financial targets, rather than a patient-centred mission, were characteristics identified in many failing organisations.^1^ High-quality interventions capable of helping struggling healthcare organisations to improve have been identified as essential.^5^ Despite extensive research on this topic, there is limited understanding of whether and how improvement interventions (intervention that seek to generate improvements in local performance) achieve effectiveness in organisational performance.^5^ Furthermore, limited attention has been paid to the negative consequences of these interventions.^4,5^

 Healthcare organisations in England rated as inadequate by the national regulator (the Care Quality Commission [CQC]) entered the Special Measures for Quality regime (SMQ) to receive increased support and oversight. The SMQ regime, a national improvement initiative, was a targeted and time-limited regime in the National Health Service (NHS) in England agreed between the CQC and NHS Improvement (NHSI). The regime emerged following the Keogh Review into avoidable mortality in 2013.^6^ SMQ originated as a programme of support and oversight targeted at NHS providers and was imported from a model of periodic inspections and increased intervention for failing schools in education.

 Healthcare organisations were put into SMQ only where serious care quality failings were identified and the leadership appeared unable to resolve the problems without intensive support and external input.^7-9^ At the time of this study, entry to SMQ remained an option if there were concerns flagged by a CQC inspection (an external regulator), coupled with a lack of confidence that the regional health system and organisational leadership is able to support the trust to make improvements. Typically, the leadership of the organisations was rated as ‘inadequate’ according to the ‘well-led’ CQC rating process and at least one other domain (ie, ‘safe,’ ‘effective,’ ‘caring,’ or ‘responsive to people’s needs’). The CQC recommended to the NHSI, by way of a letter, that a trust should be placed in SMQ since the CQC could not formally place a trust in SMQ. Discussions would then begin at NHSI about the interventions to be provided to the trust. After approximately 12 months, a re-inspection was undertaken by the CQC to ascertain whether improvements had been made and if their recommendations had been taken on board, although timelines varied. Similarly, NHSI decided when a trust was ready to exit SMQ, a decision made by the Provider Regulation Committee.

 The SMQ regime provided oversight and targeted interventions from NHSI to help organisations address specific quality failings identified in CQC inspections. There was also a ‘list’ of challenged providers (CPs) deemed to be at risk of entering SMQ that received support. Unlike SMQ, the providers on the CP list were not available in the public domain. It was informed by a number of national health agencies and regional health intelligence and was intended to serve as an early warning system to provide support to struggling health organisations at risk of entry to SMQ. Up until October 2019, 62 organisations (out of 217) were, or had been in SMQ or CP regime. NHSI interventions for healthcare organisations in SMQ/CP varied between trusts and could include all, or a combination of, the following:

- *Improvement Director (ID):* External individual equipped to support the senior leadership team. Several IDs tended to occupy senior level positions in the NHS before working in this role. There is an ‘NHSI Director cycle’ which outlines a process for providing trusts in difficulty with 1-3 months of intensive support, followed by a further three months of maintenance support.
- *Buddying:* Buddying or partnership with a well-performing healthcare organisation and commissioning of external expertise. Buddying is explained as a form of peer improvement and can be arranged directly by NHSI, by the ID or by the trust. Buddying can be on a departmental level or trust wide. Buddying is often formalised through a Memorandum of Understanding between organisations.
- *Funding:* Funding was made available to SMQ/CP trusts to deliver local quality improvements (QIs), accessible through an application made by the trust to NHSI: up to £500 000 for SMQ trusts upon entry, up to £200 000 for CP trusts, and £100 000 available to trusts upon exit of SMQ.

These interventions could be delivered in conjunction with other QI interventions, and within a context of significant senior leadership changes, including at Board level. There was limited knowledge about whether and how the NHSI interventions facilitated improvements, their implementation barriers and what balance they created between support and scrutiny. In this paper, we address these gaps by discussing the findings from an evaluation that sought to understand healthcare organisations’ experiences of being placed in SMQ/CP, variations in relation to the implementation of these interventions and the perceived impact of these interventions on QI. The study needed to generate findings rapidly to inform the future development of the SMQ/CP regimes.

###  Study Aims 

 The study focused on the three main interventions that NHSI identified as forming part of the SMQ/CP regimes and aimed to analyse the processes and responses of trusts to their implementation. The study was informed by a recent systematic review of the literature that examined the underlying concepts guiding the design of interventions aimed at low and high performing healthcare organisations, processes of implementation, unintended consequences, and their impact on costs and quality of care.^4^ This review highlighted that failure was frequently defined as the inability of organisations to meet pre-established performance standards (instead of a complex, continuously changing, situation involving internal and external factors) and turnaround was perceived as a linear process (where improvement was obtained after following a sequence of steps). Improvement interventions were designed accordingly and were focused on the organisation, with limited system-level thinking.

 Our research questions were:

1. What are the programme theories (national and local) guiding the interventions delivered to Trusts in SMQ/CP regimes?
2. What are staff perceptions of the SMQ/CP regimes and the individual interventions?
3. What are the factors that act as drivers for change when Trusts are placed in SMQ/CP?

## Methods

 Data collection and analysis were undertaken within a 13-month time frame (December 2018-January 2020) and followed a rapid research design involving teams of field researchers, co-production approaches, iterative data collection and analysis, and formative feedback. The study was classified as a service evaluation as defined by the NHS Health Research Authority, not requiring research ethics committee approval. Here we describe an overview of the primary data that supports our findings and conclusions. Further details of the methods, recruitment and data collection are available in our published protocol for a wider study on SMQ and CP^10^ and Supplementary file 1.

###  Interviews and Documentary Analysis at a National Level

 To understand the history to SMP and CP regimes and their aims, we reviewed reports and documents (n = 20) and carried out interviews (n = 6) to understand the wider regulatory and policy context, and how these had developed over time in the English NHS system.

###  Multi-site Case Studies

 Eight case studies, four ‘high level’ and four ‘in-depth’ were used to explore the implementation of interventions in SMQ/CP trusts. Case study inclusion criteria were based on NHS trusts (ambulance, acute, mental health and/or community providers) placed in SMQ and/or CP regimes before 30 September 2019. We excluded Trusts that had only been placed in Special Measures for Finance^11^ as this was not the focus of the study.

 To identify potential case study trusts, we conducted an analysis using data supplied by NHSI on 59 trusts that had entered SMQ and/or CP since the SMQ regime began in July 2013 up to 30 September 2019 and reviewed Trust CQC reports, which are publicly available. We visually plotted the performance trajectories of Trusts over time and to capture different stages of their quality journey, leading to the identification of four main groups (see also Figure):

- Trusts that enter SMQ more than once and/or have a prolonged period in SMQ of at least two years (‘prolonged poor performers’)
- Trusts that are placed on the CP ‘watch list’ and then enter SMQ (‘poor performers’)
- Trusts that are temporarily placed on the ‘watch list’ and deemed challenged, but that never enter SMQ (‘CPs’)
- Trusts that have been in SMQ and exited, going on to achieve higher CQC ratings with no re-entry into SMQ (‘clear performance improvers’).

**Figure:**
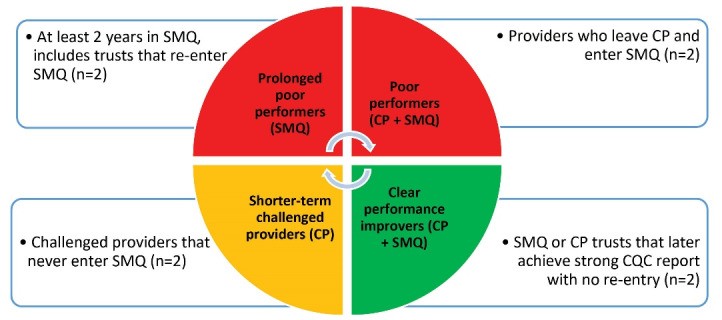
Purposive Sampling Model for Case Studies. Abbreviations: SMQ, Special Measures for Quality; CP, challenged provider; CQC, Care Quality Commission.

###  Data Collection 

 From these groups, we approached 12 sites to invite them to take part in the study, anticipating that not all sites would be interested in taking part in the study given potential sensitivities. Four sites declined the invitation, so our final sample included eight case study sites, with two sites from each performance category, a range of geographical locations, and types of trusts (see Table 1 for the site characteristics). Qualitative fieldwork combined semi-structured interviews, meeting observations (eg, public board meetings and quality committees), and documentary analysis across “in-depth” and “high-level” sites (Table 2).

**Table 1:** Characteristics of Includes Sites

**Case**	**Evaluation Performance**	**Trust Type**	**Urban/Rural/Semi-urban**
1	CP	Acute	Urban
2	Prolonged poor performer	Acute and community services	Semi-urban
3	Clear improver	Acute and community services	Semi-urban
4	CP to SMQ	Acute (teaching hospital)	Urban
5	CP	Acute and community services (teaching hospital)	Urban
6	Prolonged poor performer	Acute and community services	Rural
7	Clear improver	Acute and community services (teaching)	Rural
8	CP to SMQ	Acute (teaching hospital)	Semi-urban

Abbreviations: SMQ, Special Measures for Quality; CP, challenged provider.

**Table 2:** Summary of the Workstreams Included in the Study

**Study Element**	**Participants**	**Analysis**
**National-Level Qualitative Study**		
Interviews	6 National representatives	Understand the origins of the SMQ and CP regime, the wider regulatory and policy context, and how these had developed over time in England.
Documentary analysis	20 Documents	Understand the origins of the SMQ and CP regime, the wider regulatory and policy context, and how these had developed over time in England.
**Case Studies Qualitative Components **		
Non-participant observation (eg, board meetings, operational meetings)	8 Observations during meetings	Understand the processes used to implement the interventions as well as internal and external contextual factors influencing participation in the interventions.
Interviews	60 in the in-depth sites (from across different organisational tiers + external stakeholders) and 32 in the high-level sites (from the top of the organisation + key external stakeholders) 44 senior level participants, 13 divisional level participants and 21 external participants (for a breakdown per site, see Supplementary file 1)	Explore perceptions of being placed in SMQ or CP and the NHSI interventions, understand the processes used to implement the interventions as well as internal and external contextual factors influencing intervention participation.
Documentary analysis	291 documents	Understand the processes used to implement the interventions as well as internal and external contextual factors influencing participation in the interventions.

Abbreviations: SMQ, Special Measures for Quality; CP, challenged provider; NHSI, NHS Improvement.

 Interviews and observations were sampled purposively and used to explore perceptions of being placed in SMQ or CP and the NHSI interventions, processes of implementation and internal and external factors influencing intervention participation (see Supplementary file 1 for sampling framework). All of the members of staff we invited to take part in the study accepted this invitation.

###  Data Analysis

 Triangulation of interview, observational, and documentary data was informed by literature on change in organisations,^12-14^ a review on improvement in low-performing organisations,^4^ and literature on receptive contexts for sustaining QI in healthcare.^15^ The review shaped our analysis of failure, the implementation of improvement interventions and turnaround, as we sought to consider the complexity and continuous transformation of these processes.

## Results

 Our evaluation explored multiple dimensions of the SMQ/CP regime, including the programme theories guiding the regime and primary interventions (ID, buddying and funding), the process of implementing the interventions, and the perceptions of staff members in relation to these interventions and their impact.

###  The SMQ/CP Regimes as “Support” Programmes 

 When developing the programme theory for the SMQ/CP regimes based on the national-level interviews, we found that national stakeholders perceived the SMQ/CP regimes as “support” programmes that aimed to enable organisations to bring about improvements:

 “*Special Measures is meant to be about support from NHS Improvement, but also support from the wider system in helping them. So, it’s meant to be a helpful regime, and it’s often portrayed as a kind of punishment that isn’t the intention, but of course, it’s public, it’s reputational, I think it’s is often perceived as a punishment, but it’s not a punishment, it’s meant to be saying, ‘Actually we just don’t think, we think you need extra help in order to move yourself out of this position’” *(CQC interviewee).

 The CP regime was also viewed as a way to provide access to additional resources for struggling organisations and was not intended to be a long-term intervention. National teams recognised that providers might be part of “challenged systems” and that this needed to be taken into consideration, yet interventions were implemented at organisational level (not system level). Turnaround was perceived as a linear process, where organisations were supported until they met the standards to “leave” the SMQ/CP regimes and disregarding the potential cyclical nature of failure. In practice, several organisations returned to SM Q/CP and had to undergo a similar process of turnaround.

 Local perceptions of the SMQ/CP programme varied, with some participants seeing the programme as heavy-handed scrutiny or punishment, while others considered it “necessary,” “an opportunity and a platform to drive forward improvement” and as a “catalyst for positive change” that had a beneficial impact on the trust. There was agreement across organisations that performance issues of trusts across their patch and improvement would not be seen as sustainable until “systemic structural fault lines” were resolved. Some aspects of the programme, such as buddying, were not conceptualised as conducive to long-term improvement as system-wide changes would be needed, including changing support structures and interventions to operate at a system level as well.

###  Implementation of the Interventions

 The delivery of NHSI interventions varied across our eight case studies. In Table 3, we have identified the interventions delivered at each site and potential issues obtaining data.

**Table 3:** The Delivery of NHSI Interventions by Case

	**Case 1**	**Case 2**	**Case 3**	**Case 4**	**Case 5**	**Case 6**	**Case 7**	**Case 8**
ID		✔	✔	✔	✔	✔	✔	✔
Buddying	✔		✔			✔	✔	✔
Changes in senior management staff and/or board	✔	✔	✔	✔		✔	✔	✔
NHSI funds	✔	✔	No data^a^	No data^a^	✔	✔	No data^a^	✔

Abbreviation: NHSI, NHS Improvement. ^a^Trust was not able to supply data.

####  Improvement Directors

 IDs were appointed by NHSI when deemed necessary for a Trust in SMQ or CP. The normal cycle for an ID normally included 1-3 months of intensive support, followed by three additional months of maintenance support (although this might vary by Trust). Some IDs highlighted that a time limit on the time they were involved with organisations was required: *“Personally I think you lose your effectiveness as an Improvement Director when you’ve been in an organisation for about eight to ten months*” (ID 1). However, this feeling was not always shared with Trusts as some felt IDs left when important work was still to be done: “*Our ID was withdrawn last summer, long before we had been reinspected or let alone, come out of special measure, so I employed one of my former IDs to help us, […] but the Trust had to fund that itself*” (CEO, case 2). Interviews at Trusts level revealed that QI Plans were a central element of SMQ/CP regimes and an essential role of IDs who would engage senior organisational leaders and support the development of an improvement strategy and vision for the organisation. IDs would often cover more than one Trust at the same time and some Trusts in CP might not have access to an ID. They were a limited resource.

####  Buddying

 Buddying, also referred to as peer improvement, could be arranged directly by NHSI, by the ID or by the Trust themselves. Buddying could be on a departmental level – if specific improvements were required in one service area – or organisation-wide. Buddying was often formalized through a Memorandum of Understanding and (well performing) buddying organisations were sometimes paid for supporting a poor-performing provider in the SMQ regime. In the case of our study, we were only able to identify cases of buddying in five Trusts. The use of buddies varied by Trust, and most Trusts used them to learn about good practices in relation to specific problems where they needed to bring about QI. The appropriateness of buddies was discussed frequently and the first selection of buddies made by NHSI was not always considered a good choice by Trusts in SMQ, which is why some organisations arranged their own buddies: “*They are a large metropolitan university teaching hospital and we are not and it just wasn’t compatible and wasn’t really working and the improvement work that they were doing with us was focused on things we didn’t need immediate help with*” (CEO, case 2). The appropriateness of the matching was dependent on geographic location (with close distance seen as positive), Trust type and size and having successfully tackled similar performance problems.

####  Funding

 Trusts in SMQ and CP had the opportunity to apply for and, if successful, access funds to help support improvement activities. Trusts labelled as CP were able to apply for up to £200 000 and Trusts in SMQ for £500 000. Trusts also received additional financial support when leaving SMQ (£100 000). Applications for the funds are reviewed internally at NHSI and approved by the Executive Medical Director: “[*the application] needs to be mapped to things that are going to make a difference and […] how it has impacted and made a difference*” (NHSI representative). Six Trusts in our study had access to the support funds provided by NHSI. The remaining two Trusts did not have access to funding as their period in SMQ pre-dated the financial support component of the regime. The funds were mainly used to cover the costs of external consultants, organizational development and to fund posts. In some cases, Trusts expressed concern they would need to “spend their way out of special measures.” The funds were not considered by respondents to be enough to support long-lasting QI.

###  Additional Interventions

 In addition to the core interventions of IDs, buddying and funding, there were other NHSI led interventions that were frequently mentioned by participants; including “deep dives” and risk assurance through additional system oversight and scrutiny. Four of the case study sites mentioned deep dives that they had carried out with NHSI staff through intensive analysis of data and information on specific topics/service areas. Examples included a review of emergency department performance, staff engagement through focus groups to identify areas for improvement and developing of infection monitoring strategies at ward level. Additional risk assurance could include reporting to specific organisations such as Clinical Commissioning Groups (CCGs) or the use of Oversight and Assurance Groups (OAGs), which were formed to oversee the Trust’s progress and provide assurance on the delivery of the CQC recommendations. OAG members were drawn from key external stakeholder groups, such as the CQC, CCG, Healthwatch and Health Education England. The additional risk assurance processes were seen as potentially good in theory, but could be burdensome in practice due to time and resources required to gather data for reporting, simultaneous demands and unrealistic timelines.

###  Staff Perceptions of SMQ/CP and the Individual Interventions

 The SMQ/CP regime could be viewed positively by respondents, especially in hindsight when trusts had gone on to sustain improvement, with some organisations feeling they received the right support, were allowed space to deliver QI, and that the organisation needed external challenge due to recalcitrant cultural and performance issues. However, others saw SMQ as heavy-handed scrutiny or punishment, particularly in the short term when a poor CQC inspection report and quality rating created reputational damage and could impact on staff morale and recruitment. Over time, there was often a shift to a more positive view of SMQ/CP as a needed catalyst for positive change. Over time, most staff appreciated that the SMQ/CP regime gave the organisation time to focus on quality and address issues that had not been tackled for a long time (eg, bullying culture). However, we found variability in the perceptions of the specific improvement interventions within and between Trusts. For instance, some staff identified the value of the ID, while other members of staff in the same organisation did not agree with this role.

 The perceptions of NHSI interventions of IDs, buddy organisations, and funding were mixed overall. Trusts had individual issues and needs for support, requiring specific tailoring of the interventions. IDs were considered helpful when using a coaching style and offering tactical advice for interacting with a complex system of health regulators and external agencies, each with their own reporting requirements: “*Working with my current [ID], we sat down and I said, ‘Look, I’ve already appointed a Programme Director. This is what I’m going to do. This is how I’m going to do it. This is the governance structure within which I will work. Can you start with the board, because of Well-Led being rated inadequate?’ She started to deal with the stuff around the board conversations, which was really helpful to me because I then could get on [with QI]*” (Clinical Director). IDs were sometimes felt to bring additional demands on organisations and there were mixed views about the amount of time IDs should spend in organisations – especially to avoid fostering dependency on outsiders. Buddies were most commonly used to learn about good practice in relation to specific problems; the partnership worked best when ‘buddy’ organisations had similar contexts.

 Additional funding was reported as being mainly used to cover posts and external consultants. Some participants felt there was a risk that trusts might need to spend their way out of SMQ. Changes to leadership teams which occurred in parallel could bring beneficial new ideas and bring new perspectives to existing problems with organisational culture. However, stability of leadership was equally critical rather than a revolving door of CEOs which was a risk for Trusts in Special Measures. In four case study trusts, the new leadership teams included people with previous SMQ knowledge, who provided first-hand experience and insights about delivering QI and responding to regulatory systems which generated confidence amongst senior staff:

 “*I knew what [funding] I was going after. So, I sat down with the [finance team] as soon as I got here and said, ‘Right, this is how we’re going to do it. This is what we’re going to go for’… I’d already got the report ready for our ID to put in for my financial support for my quality improvement faculty. So, I know where I’m going. So, I think that helps, because I know the post, because it helps me negotiate it quickly… And I guess I can navigate, very easily, the regulatory conversation about the organisation, so that makes it easier for me*” (Clinical Director).

###  The Unintended Consequences of SMQ

 There was an emotional impact on staff of their organisation being labelled as failing and placed in SMQ/CP. For example, staff across the organisation were described as being “*shocked*,” “*devastated*,” “*angry*,” “*ashamed*” and “*mortified*” when first faced with entering SMQ/CP. The stigma of the SMQ label contributed to the lowering of staff morale, exacerbating existing problems with the recruitment and retention of staff, and negatively impacting on how the organisation was viewed and treated by local peers and partners. Duplicate reporting, overwhelming workloads and stress were also seen as a result of SMQ/CP. Participants felt that SMQ places *“enormous pressure on senior staff” *and has an *“impact on people’s well-being and health.”* The regulatory requirements from the CQC and other organisations requesting reports on progress and quality assurance needed large amounts of time and resources to gather information and data: Notably, one ID mentioned, “*It should be helpful, but isn’t. It requires a lot of work to ‘feed’ the OAG each month.*” Another unintended consequence of SMQ was the negative impact on the healthcare organisation’s capacity to participate in local collaborations due to the time and focus required internally to address for regulatory requirements. Accordingly, some participants described a “*tension*” between having the capacity to deliver QI, and being able to contribute to the wider system.

###  Factors That Acted as Drivers for Change

 We found that healthcare organisations focused their improvement activities across four domains (Table 4) which are not mutually exclusive. Healthcare organisations prioritised areas for improvement in light of CQC inspection reports and regulatory recommendations, often with strategic support from an ID and sometimes external advisors (eg, NHSI, CQC or management consultancies).

**Table 4:** Organisational Processes That Acted as Drivers for Change

**Domain**	**Examples From the Case Studies**	**Illustrative Quotes**
Improving governance arrangements	Review of governance and accountability; increased “board to ward” interactions; development of sustainable strategies for QI and patient safety.	*“We visited 90 areas across the trust within a three-month period after the last report came out and drew up findings, reports, action plans and then we repeated it three months later and we're now on our third iteration of walkabouts. So, by the end of the year, every area in the hospital, whether it's clinical or non-clinical, will have been visited at least once by a team consisting of a non-executive director, a senior manager or an executive and a lay partner. Either a governor or a patient” *(Non-executive director, case study trust).
Developing clinical leadership	Better clinical leadership at senior, divisional and ward levels.	*“Evidence of serious deficits in leadership in a series of ways, both through the sort of feedback from staff about lack of staff engagement, not being listened to, bullying and harassment allegations and so on, but also clear evidence of very little clinical leadership in the organisation, so what we have focused on really is trying to get leadership culture and morale and staffing right, seeing them as the underlying themes” *(Senior director, case study trust).
Staff engagement	Address problems with organisational culture (eg, bullying); recognise and celebrate staff; improve lines of communication between senior team and staff.	*“If you want to create the right environment to change and to improve things, you’ve got to invest in the people” *(Senior director, case study trust).
External partnerships	Understand ‘failure’ across the system, develop collaborative partnerships with other organisations.	*“Many of the solutions to the problem of the acute hospital lie outside the hospital” (Senior director, case study trust).*

Abbreviation: QI, quality improvement.

####  Improving Governance Arrangements

 Many trusts focused on improving their governance and assurance processes. Better leadership visibility came about through increasing “board to ward” interactions and having members of the senior leadership team communicating more frequently with frontline staff, undertaking ward visits, and ensuring there were clearer lines of accountability with greater opportunities for senior leaders to listen to the concerns of staff.

####  Building Clinical Leadership

 There was recognition across sites for the need to ensure there was excellent clinical leadership at divisional and ward levels, as well at the top of the organisation, to bring about improvements; that frontline clinical staff also needed to understand why specific improvements or changes in process were necessary and be engaged in delivering and leading improvements at the local level. In some sites, for example, changes in local emergency department leadership were encouraged to bring about improvements to accident & emergency performance. Part of the journey of improvement was ensuring that, at all organisational levels, there was leadership accountability for managing risk, making improvements and embedding processes for assurance. Some of the participants highlighted the contributions made by Quality Committees and having engaged staff presenting at Board and Division meetings.

 In terms of senior level oversight, the Medical Director and Chief Nursing roles appeared vital for reconnecting divisional and senior executive leadership tiers, ensuring that clinical engagement was Trust-wide. The combined effect of these roles at the apex of the organisation – chief executive officer (CEO), Doctor of Medicine (MD), Chief Nurse – may have been overlooked in previous research that has focused on the transformational impact of hospital CEOs and single leaders as opposed to senior leadership teams that balance clinical and managerial input. Another activity that Trusts were required to enact was filling vacant senior leadership posts. Several Trusts had struggled to recruit and relied on interims which was potentially destabilising for the organisation as a whole or resulted in notable leadership gaps, such as where it was difficult to recruit a lead nurse.

####  Staff Engagement

 Culture change was closely intertwined with senior leadership and improving staff engagement. Arguably, culture change could only begin with improving relations amongst staff and bringing staff along an improvement journey and vision set out by the Trust’s leaders. Examples of attempts to better staff engagement included: focus groups with staff, with NHSI support and workshops with staff. Attempts at proactive engagement with staff by new leaders was a common theme across the case studies. To improve staff engagement, the Board, senior executive team and managers become more “visible, supportive and approachable.” Staff at one site, for example, observed that the CEO and team were often seen and used social media (eg, Twitter) to communicate with staff and keep them informed. Many interviewees described that, previously, there had been a gap between the senior executive team and frontline staff and poor communication.

 Measures taken to tackle historical issues with poor staff survey results and engagement include encouraging a culture of openness and transparency. There was evidence of senior leaders celebrating staff successes, and especially amongst the clear improvers case studies. Several sites made efforts to improve events and facilities for doctors and trainees (eg, “new consultants day”) and improvements were demonstrated through a higher number of junior doctors applicants achieved. Finally, there was evidence of improving sites encouraging staff not only to complete mandatory training and appraisals, but to progress their careers within the Trust and develop new knowledge through QI training.

####  External Partnerships

 In several Trusts, it was evident that there were systemic problems within the local region, and it was noted, both at national level and in the case studies, that local system-wide issues may need to be addressed for a trust to exit SMQ/CP and sustain improvements in quality and performance over time. In addition, being placed in SMQ/CP could result in improved system-wide relationships and encourage collaborative working between organisations. However, entry into SMQ/CP did not always lead to improved relationships as examples of continued regional problems were also seen.

## Discussion

 Our rapid evaluation adds new empirical knowledge on the implementation of national improvement initiatives delivered using centralised top-down approaches to failing hospitals. The programme theories guiding the design and implementation of the SMQ/CP programme at a national scale depicted it as a supportive intervention that aimed to provide organisations with the funding, expertise and tools to enable improvements. At a local level, views ranged from seeing the programme as providing much needed local support and space for reflection to its representation as punitive and burdensome in organisations that were already under significant pressure.

 Instead of a ‘one size fits all’ model, we found that external strategies to support improvement need to be more trust specific and consider ways to mitigate the emotional cost and stigma of SMQ/CP. These trust-specific improvement strategies can make use of the organisation’s tacit knowledge on their needs and improvement interventions that might be more appropriate for their local context. A summary of the key lessons from our study is provided in Box 1. Contrary to studies that have not identified the positive aspects of top-down policy directives,^16^ our study found the benefits of a combination of external scrutiny with dedicated time and resources for the organisation to focus specifically on quality and service improvement (as opposed to financial control). This could be an effective strategy for organisational improvement provided that the NHSI interventions were tailored to the organisation’s needs and local context; there was sufficient time to embed the changes required; and a well-functioning leadership team and Board.

**Box 1. Key Lessons From the Study for Trusts and Regulators and Wider Literature**

- Ways to mitigate the emotional cost and stigma of SMQ are needed.
- Time is needed to implement and embed sustainable changes and staff should be given ‘slack’ to develop and implement changes.
- Strategies to support improvement need to be organisation specific.
- Reduce duplication of reporting requirements to different regulatory bodies.
- Poor organisational performance needs to be considered at organisational and system levels (considering the factors that might be hindering improvement at system level).
- There is a need for the stability of leadership to turnaround organisations because of the amount of time improvement takes – otherwise problems are perpetuated.
- Inclusion of people with previous experience with SMQ in senior leadership teams can help manage regulatory requirements and bring knowledge and confidence to enacting change.
- Development of organisational-wide QI strategies and capabilities is important.
- Staff engagement and an organisational culture that supports learning are key to sustainable improvement.
- Trusts in SMQ/CP need support from other organisations in their local system.

---------------

Abbreviations: SMQ, Special Measures for Quality; CP, challenged provider; QI, quality improvement.

We also found that staff need ‘slack’ to develop and implement changes and to develop internal QI capabilities and capacities, such as training staff on new QI tools and methods.^15^ Jones et al,^15^ have also noted that organisational improvement is a long journey and it can be difficult to maintain momentum. This finding is consistent with findings from the literature that have argued that protected staff time is required for implementation of improvement interventions, clear priority-setting and the use of routine data to monitor progress at Board level.^17-22^ Further, others have emphasised the importance of supporting the development of organisational learning capacity to address performance failure.^23^

 As others have found, the development of organisational-wide QI strategies that encourage high levels of staff engagement, and an organisational culture that supports learning, are key to sustainable QI.^5^ Our study highlighted that senior leadership teams (rather than an individual leader) were seen as a driver for improvement, providing they were highly engaged with and visible to staff and in post for a substantial period of time. We found that changes to senior leadership teams, and the inclusion of people with previous experience with SMQ can help to enact positive change, but, at the same time, there was a need for stability of leadership to ensure continuity of the implemented approaches. The roles of the Medical Director and Chief Nurse were critical, resonating with trends in the literature that have highlighted that CEOs alone may have a limited impact on hospital performance overall,^24^ but “triumvirate” approaches (CEO, MD, Chief Nurse) to leadership can be valued for supporting patient-centred care and QI.^20,25-27^ A senior team that invested in QI and remained supportive of staff could lead to sustained improvement, and achieve turnaround in organisations that had troubled pasts (eg, problems with bullying and maintaining performance standards).

 Previous research in the area of organisation improvement has done little to address the role that wider local healthcare systems play.^4^ Our review on improvement interventions delivered to ‘low’ and ‘high’ performing organisations also pointed to intervention models that focused on the factors influencing performance at an organisational level, disregarding how these could be shaped by external factors.^4^ Our study found that poor organisational performance needed to be considered at both organisational and system levels: local healthcare systems and peer organisations could contribute to performance improvement through integrated care models and collaborative relationships. Shifting the improvement focus to a system or regional level may promote sustainable improvement over the long-term. Furthermore, turnaround needs to be considered a complex and iterative process and linear models of improvement need to be avoided. This has been recognised by the NHS Long Term Plan,^28^ (encouraging collaboration between providers) and new operating models for oversight (placing emphasis on system working).^29^

 We found negative consequences of SMQ/CP; the stigma of the SMQ label contributed to the lowering of staff morale, exacerbated problems with recruitment and retention, and negatively impacted on how the hospital was perceived by local partners. Recruitment and retention difficulties for trusts in SMQ, strain on management systems and lower staff and patient morale were also noted by in a commentary by Rendel et al^30^ and have been discussed in the education sector.^31^

###  Strengths and Limitations of the Study

 This is the first study of the response to, and impact of, the SMQ/CP regime. The key strength of the study is the case study design, which has allowed us to look at eight NHS Trusts and capture perceived local impacts on staff and internal improvement processes. The one-year timeframe has meant that the longitudinal study of change in the case studies was limited, although retrospective interviews and analysis of board papers and CQC reports offered a longitudinal view of internal trust issues and how they were tackled over time. In addition, some data were retrospective and changes in policies have occurred over the course of the study period. It is also possible that access to case study sites was constrained due to the sensitive nature of the research topic. As we reported earlier in the paper, some of the sites that declined our invitation to take part in the study could have different experiences with the SMQ/CP regime than those that accepted our invitation.

## Conclusion

 Supporting poor performing healthcare organisations to improve is essential and we have added to the limited knowledge base on the implementation and impact of improvement interventions by focusing on processes of implementation, unintended consequences and perceived impact. Future research should focus on the evaluation of the impact of improvement initiatives that include a greater focus on involving local systems. This could be achieved through the use of sequential monitoring techniques to allow “real-time” assessments of the impact of interventions, prospectively linking financial stability to changes in direct/indirect costs and additional opportunity costs using indicators that are part of routinely reported data. Future longitudinal studies should also look at the sustainability of improvement.

## Ethical issues

 The UCL R&D Office and Ethics Committee reviewed the study protocol and materials. The study was classified as a service evaluation as defined by the NHS Health Research Authority (HRA), not requiring research ethics committee approval. Guidelines for data security, confidentiality and information governance have been followed. An informed consent process using participant information sheets and written consent was used for recruitment to ensure informed and voluntary participation. We are aware of the sensitive nature of this research for organisations and individuals. The research team has experience in conducting research on similar sensitive topics. The independence of the research and the anonymity of participants and organisations has been upheld.

## Competing interests

 Authors declare that they have no competing interests.

## Authors’ contributions

 NJF, CVP, JL, MH, ST, and JS contributed to the conception and design of the study. MH, JL, and CVP contributed to data collection for the qualitative study. CVP led on the drafting of the manuscript. All authors contributed to revision of the manuscript and approved the final manuscript.

## Funding

 This manuscript is independent research funded by the NIHR (Health Services and Delivery Research, 16/138/17 – Rapid Service Evaluation Research Team). NJF is an NIHR Senior Investigator.

## Disclaimer

 The views expressed are those of the authors and not necessarily those of the NHS, the NIHR or the Department of Health and Social Care.

## Supplementary files

Supplementary file 1. Details of the Qualitative Study.
