# Supplementary material for: The Special Measures for Quality and Challenged Provider Regimes in the English NHS: A Rapid Evaluation of a National Improvement Initiative for Failing Healthcare Organisations
Source: Int J Health Policy Manag. 2022 Apr 27;11(12):2917–26. doi: 10.34172/ijhpm.2022.6619 (PMC10105181; doi:10.34172/ijhpm.2022.6619)
Supplement: Supplementary file 1 — Details of the Qualitative Study. [file ijhpm-11-2917-s001.pdf]

Article title: The Special Measures for Quality and Challenged Provider Regimes in the English NHS: A Rapid Evaluation of a National Improvement Initiative for Failing Healthcare Organisations

Journal name: International Journal of Health Policy and Management (IJHPM)

Authors' information: Cecilia Vindrola-Padros[1,2]*, Jean Ledger[1], Melissa Hill[3], Sonila Tomini[1], Jonathan Spencer[4], Naomi J. Fulop[1]

[1]Department of Applied Health Research, University College London, London, UK.

[2]Department of Targeted Intervention, University College London, London, UK.

[3]NHS North Thames Genomic Laboratory Hub, Great Ormond Street Hospital, London, UK.

(*Corresponding author: c.vindrola@ucl.ac.uk)

Supplementary file 1. Details of the Qualitative Study

Types of NHS trust entering SMQ or CP regimes (July 2013-September 2018)

**Trust type**	**Number of trusts ever in the SMQ or CP regimes**	**Trusts in SMQ (at September 2018)**	**Trusts in the CP regime (at September 2018)**
Acute services only	33	7	9
Acute and community	18	4	7
Acute and mental health	1	1	0
Ambulance	2	1	0
Community and mental health	1	0	1
Mental health	4	1	0
TOTAL	59	14	17

Data collected for the qualitative components of the evaluation

**Data source**	**Case**								**Totals**
	**1**	**2**	**3**	**4**	**5**	**6**	**7**	**8**	
Senior Team Interviews	6	4	6	6	6	4	6	6	**44**
Divisional Level Interviews	5	2	3	2	-	1	-	-	**13**
External Interviews	2	2	4	4	2	-	3	4	**21**
Total Interviews	13	8	13	12	8	5	9	10	**78**
Total meeting observations	2	-	5	2	-	2	-	-	**11**
Documents	27	29	71	33	52	55	14	10	**291[a]**

[a] This is a minimum estimate based on the main documents used for analysis. Additional documents were reviewed and trust websites accessed. Particularly helpful documents for analysis included public trust board minutes and associated papers; Quality Committee minutes and papers; trust performance reports; QI plans and resources; CQC inspection reports; NHSI letters to trusts.
